# Insulin-like growth factor-II mRNA-binding protein 3 predicts a poor prognosis for colorectal adenocarcinoma

**DOI:** 10.3892/ol.2013.1458

**Published:** 2013-07-12

**Authors:** LIJUAN LIN, JINHUI ZHANG, YAN WANG, WEIEI JU, YIBING MA, LINA LI, LITIAN CHEN

**Affiliations:** 1 Department of Medicine Imaging, Eastern Liaoning University College of Medicine, Liaoning 118000, P.R. China; 2 Department of Pathology, Dandong Centre Hospital, Dandong, Liaoning 118000, P.R. China; 3 Department of Pediatrics, Tianjin First Centre Hospital, Tianjin 310092, P.R. China; 4 Department of Liver Transplantation Surgery, Xin Hua Hospital, Shanghai Jiao Tong University School of Medicine, Shanghai 200092, P.R. China

**Keywords:** colorectal adenocarcinoma, insulin-like growth factor-II mRNA-binding protein 3, immunohistochemistry, prognosis

## Abstract

Insulin-like growth factor-II mRNA-binding protein 3 (IMP3) has been recently identified as a marker of aggressive behavior in several types of tumors. The aim of the present study was to detect the expression of the IMP3 protein in colorectal adenocarcinoma (CRA) and to identify a correlation with the clinicopathological features of the disease. IMP3 was evaluated in 186 samples of CRA using immunohistochemical methods. The correlation between IMP3 expression and the clinicopathological features of colorectal cancer was evaluated by the χ^2^ and Fisher’s exact tests. Survival rates were calculated using the Kaplan-Meier method and the correlation between IMP3 protein expression and the prognosis of patients with CRA was analyzed using Cox analysis. Of the 186 adjacent normal mucosa (ANM) cases, the 22 that exhibited dysplasia demonstrated weak IMP3 expression and the 164 without dysplasia showed no expression. Of the 186 CRA cases, immunohistochemical staining for IMP3 was observed in 143 cases (76.9%). A comparison of IMP3 expression between the CRA and ANM samples revealed stronger immunohistochemical reactivity in the CRA tissues (P<0.01). High IMP3 expression was associated with differentiation, lymphoid metastasis, TNM stage, Ki-67 labeling index and a poor patient outcome (P<0.05). In the multivariate analysis, IMP3 emerged as an independent predictor of survival. The present study demonstrated that IMP3 is able to promote the aggressiveness of cancer behavior, resulting in a poor prognosis for patients with CRA. Consequently, IMP3 may be regarded as a novel proliferation and prognostic indicator for patients with CRA.

## Introduction

Colorectal cancer is the most common malignancy of the gastrointestinal tract (1) and causes 655,000 mortalities worldwide every year (2). As it has high recurrence and metastasis rates, there is an urgent requirement to identify specific markers that are closely associated with the bionomic characteristics of colorectal adenocarcinoma (CRA), the outcome of affected patients and the performance of an antigen-specific therapeutic targeting strategy.

Insulin-like growth factor-II mRNA-binding protein 3 (IMP3), an oncofetal protein and member of the IMP family, has become a focus of attention as it appears to play a significant role in cell migration and adhesion in various malignant neoplasms (3). IMP3 is a 580-amino acid protein with four K-homology domains and two RNA recognition motifs. The protein is encoded by a gene on chromosome 7p11.5 (4) and has been known in previous studies as the K-homologous domain-containing protein that is overexpressed in cancer. IMP3 is also known as L523S, a regulatory binding protein believed to be involved in the stabilization and intracellular trafficking of IGF-II mRNA to facilitate IGF-II production (5). IMP3 is expressed in a number of the cells of a developing fetus, but is absent in the majority of adult cells, with the exception of the gonads. The overexpression of IMP3 has been identified in a number of malignant tumors, including renal carcinoma (6), malignant pancreatic lesions (7), endometrial carcinoma (8), uterine cervical cancer (9) and testicular cancer (10).

However, to the best of our knowledge, the expression of IMP3 in CRA has rarely been studied. In order to further determine the role of IMP3 in neoplastic pathology, the present study evaluated the expression of IMP3 in CRA using immunohistochemical techniques. The present data aimed to reveal the correlations between IMP3 expression and the clinicopathological features of CRA in order to determine whether the expression of the IMP3 protein may serve as a biomarker for the prognostic evaluation of CRA

## Materials and methods

### Patients and tissue samples

Tumor specimens were obtained from 186 patients (119 males and 67 females; mean age, 59.3 years; range, 25–82 years) who underwent surgery for CRA between January 2004 and May 2007. All the adjacent normal mucosa (ANM) tissues from the cancer resection margins were also included and none of the patients were administered prior chemotherapy or radiotherapy. The pathological parameters, including patient age, gender, tumor grade, nodal metastasis, clinical stage and survival data, were carefully reviewed in all cases. The HE stained slides were reviewed by two experienced pathologists and one appropriate paraffin block with tumor and ANM tissue was selected for this study. Informed consent was obtained from each patient prior to conducting the study and approval for the study was obtained from the Ethics Committee of Dandong Centre Hospital (China).

### Immunohistochemistry for IMP3 in paraffin-embedded tissues

For the immunohistochemical study using the Dako labeled streptavidin-biotin (LSAB) kit (Dako A/S, Glostrup, Denmark), 4-μm thick tissue sections were deparaffinized, rehydrated and incubated with 3% H_2_O_2_ in methanol for 15 min at room temperature (RT), in order to eliminate endogenous peroxidase activity. The antigen was retrieved at 95ºC for 20 min by placing the slides in 0.01 M sodium citrate buffer (pH 6.0). The slides were then incubated with primary antibodies (polyclonal goat antiserum for IMP3; dilution, 1:150; N-19; Santa Cruz Biotechnology, Inc., Santa Cruz, CA, USA) and monoclonal mouse antiserum for Ki-67; MAB-0129; Maixin Technology Co., Ltd., Shenzen, Guangdon, China) at 4ºC overnight. Subsequent to being incubated at RT for 30 min with biotinylated secondary antibody, the slides were incubated with streptavidin-peroxidase complex at RT for 30 min. Immunostaining was developed using chromogen and 3,3′-diaminobenzidine and then counterstaining with Mayer’s hematoxylin. Goat IgG isotopes, which showed a negative staining result, were used as controls,. The positive tissue sections were processed omitting the primary antibody and were used as negative controls.

### Evaluation of immunohistochemical staining

A positive stain for IMP3 was defined as a brown stain observed in the cytoplasm, and for Ki-67, as a stain observed in the nucleus. All specimens were examined by two pathologists who did not possess prior knowledge of the clinical data. In the case of discrepancies, a final score was established by reassessing the slides on a double-headed microscope. A lack of staining for IMP3 was scored as - and the intensity of positive staining was evaluated as weak, + or strong, ++ (11). The immunostaining for Ki-67 was scored as - (negative, none or ≤5% positive cells) and + (positive, >5% positive cells).

### Statistical analysis

The statistical analyses were performed using SPSS 17.0 software (SPSS, Inc., Chicago, IL, USA). The correlation between IMP3 expression and the clinicopathological characteristics was evaluated using the χ^2^ and Fisher’s exact tests. The correlation between IMP3 protein expression and Ki-67 was evaluated using Spearman’s correlation analysis. The survival rates following the removal of the tumor were calculated using the Kaplan-Meier method and the difference between the survival curves was analyzed by the log-rank test. A multivariate survival analysis was performed on all the significant characteristics that were measured by the univariate survival analysis through the Cox proportional hazard regression model. P<0.05 was considered to indicate a statistically significant difference.

## Results

### IMP3 protein expression in various colorectal mucosae

**Figure 1:**
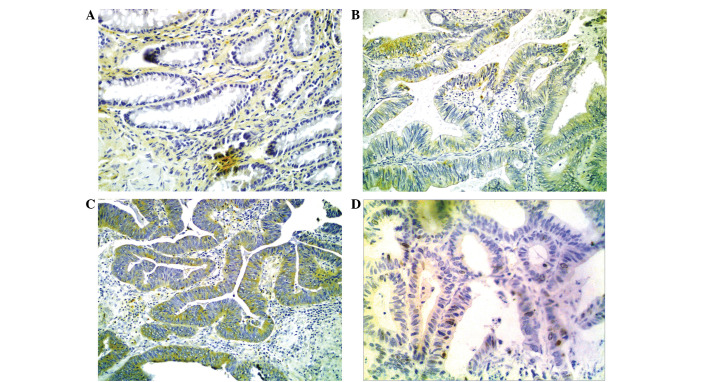
Immunohistochemical staining. (A) No immunoreactive staining of IMP3 protein in the ANM (×200). (B) Cytoplasmic expression (weak, +) of IMP3 in CRA tissues (×200). (C) Cytoplasmic expression (strong, ++) of IMP3 in CRA tissues (×200). (D) Nuclear expression of Ki-67 in CRA tissues (×400). IMP3, insulin-like growth factor-II mRNA-binding protein 3; ANM, adjacent normal mucosa; CRA, colorectal adenocarcinoma.

Of the 186 ANM cases, the 22 that exhibited dysplasia demonstrated weak IMP3 expression and the 164 without dysplasia showed no expression. Of the 186 CRA cases, immunohistochemical staining for IMP3 was identified in 143 cases (76.9%). The immunohistochemical reaction intensity for IMP3 was identified to be weak in 82 cases (44.1%) and strong in 61 cases (32.8%; Fig. 1). The expression of IMP3 in the CRA tissues was significantly stronger than in the ANM tissues (χ^2^=49.183, P<0.001; Table I).

### Correlation between IMP3 protein expression and clinicopathological factors

A higher IMP3-positive rate was detected in the cases of CRA with lymphoid metastasis compared with those without lymphoid metastasis (94/111 vs. 49/75; χ^2^=9.430; P=0.002). Additionally, there was a significant difference in the TNM stage between the CRA tissues with IMP3 expression and those without (P=0.001; Table II). The tumors at higher stages showed an increased level of IMP3 expression. It was indicated that IMP3 expression was not significantly correlated with age, gender, size, histological grade or carcinoembryonic antigen (CEA) level (P>0.05; Table II).

### Correlation between IMP3 protein expression and Ki-67 expression in CRA

As shown in Fig. 1, the immunoreaction of Ki-67 was localized in the nucleus. The positive Ki-67 protein expression rate was 54.8% (102/186) in the CRA tissues. Positive IMP3 gene expression was strongly associated with the Ki-67 labeling index (r=0.169; P=0.021; Table III).

### Correlation between IMP3 protein expression and prognosis

**Figure 2:**
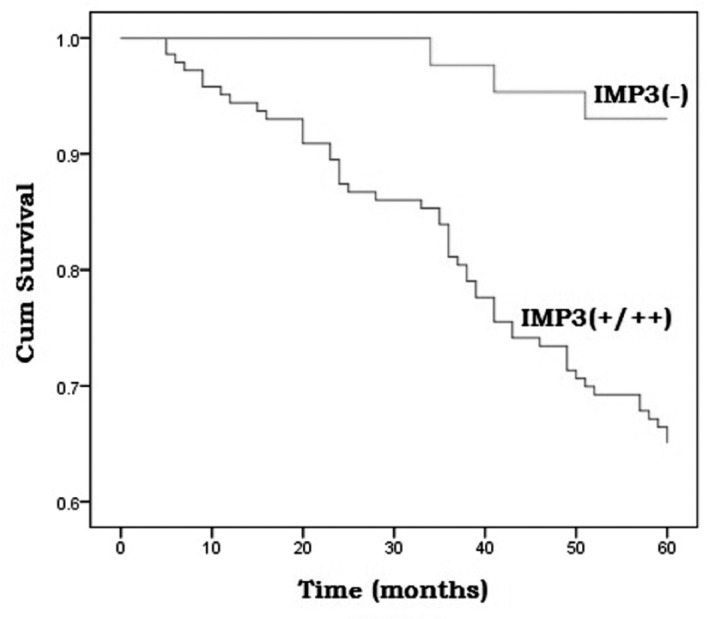
Kaplan-Meier analysis of post-operative survival in patients whose tumors lacked IMP3 expression (−; n=43) vs. those with positive expression (+/++; n=143). Patients with positive IMP3 expression had a shorter survival than those lacking expression (log-rank=11.775, P=0.001). IMP3, insulin-like growth factor-II mRNA-binding protein 3; cum, cumulative.

To further confirm the role of IMP3 expression in CRA progression, the survival rates of the 186 CRA cases were analyzed using the Kaplan-Meier method. The cases that demonstrated positive IMP3 staining (+/++) had a lower survival rate than those that were negative for IMP3 immunoreactivity (P=0.001; Fig. 2). The multivariate analysis was performed using the Cox proportional hazards model for all the significant variables in the univariate analysis. IMP3 was identified as an independent prognostic factor in colorectal cancer (HR, 0.618; 95% CI, 0.394–0.972; Wald χ^2^=4.343; P=0.037).

## Discussion

The IMP3 gene was originally identified from a pancreatic tumor cDNA screen in 1996 (12) and was subsequently cloned. IMP3, together with IMP1 and 2, are members of the human IMP family that were first purified from the human rhabdomyosarcoma cell line, RD, in 1999 (13). IMP3 has since been shown to be expressed in a number of solid tumors and fetal tissues. IMPs have been reported to play a pivotal role in the binding, trafficking, stabilization, growth and migration of cells during embryogenesis (3). IMP3 regulates the gene expression of IGF-II by binding to its 3′-mRNA region. IGF-II then binds to and activates IGF-I while stimulating the tyrosine phosphorylation of this receptor. The tyrosine phosphorylated IGF-I receptor transmits mitogenic signals to the cell. This is followed by cell cycle regulation loss and apoptotic cycle disturbance, resulting in uncontrolled cell proliferation and carcinogenesis (14,15). Therefore, it is hypothesized that IMP family members are involved in carcinogenesis by stabilizing IGF-II mRNA. However, the IMP proteins are also able to bind and affect other mRNAs, which may affect the malignant potential of cells.

The present study aimed to determine whether the expression of the IMP3 oncoprotein may serve as a biomarker for the prognostic evaluation of CRA. According to the present results, IMP3 was expressed in the carcinoma lesions, but was almost absent in the adjacent tissue counterparts. Similar associations have been demonstrated between IMP3 expression and older age, larger tumor size, deep tumour invasion and lymph node metastasis (16).

Ki-67 is an established marker of cell proliferation that correlates with the progression of the cell cycle and that is expressed in G_1_, S, G_2_ and mitosis (17). In the present study, Ki-67 was selected to represent the proliferation status of the cells and the Ki-67 labeling index was shown to be significantly correlated with IMP3 expression. Studies have shown that the oncofetal protein, IMP-3, appears to play a critical role in arranging cellular proliferation, tumor invasion and aggressive behavior (18,19). The present data also suggested that the IMP3 protein was a significant proliferation marker for tumor cells.

Yaniv *et al*(20) reported that IMP3 in *Xenopus laevis* is required for the migration of cells that form the roof plate of the neural tube and, subsequently, for neural crest migration, suggesting that IMP3 is important for promoting cell migration. The expression of IMP3 in tumor cells has been associated with an unfavorable outcome in renal clear-cell carcinoma (7,21). In the present study, IMP3 was shown to be highly expressed in CRAs with lymphoid metastasis compared with non-metastatic tumors (χ^2^=9.430; P=0.002). The correlation between IMP3 expression and lymphoid metastasis implies that IMP3 may promote lymphoid metastasis in CRA. Furthermore, significant differences in the TNM stages were observed between the CRA tissues that expressed IMP3 and those that did not.

Survival analyses have indicated that IMP3 expression is negatively linked to a favorable prognosis for gastric adenocarcinoma (18,22), renal cell carcinoma (6) and hepatocellular carcinoma (23). IMP3 expression has been shown to significantly affect the five-year survival rate of patients with CRA. The patients with IMP3-positive immunoreactivity had significantly shorter survival times compared with those who were negative for IMP3. These findings and those of other studies may indicate a correlation between IMP3 expression and aggressive tumor progression and metastasis.

In conclusion, IMP3, a novel oncofetal mRNA-binding protein, is frequently expressed in CRA. IMP3 expression is more commonly seen in cases with poor prognostic factors of CRA, leading to lymphoid metastasis, late-stage cases and short survival times. Immunohistochemistry for IMP3 may be a potential biomarker to evaluate the tumor progression and prognosis of CRA.

## Figures and Tables

**Table I:** IMP3 protein expression in ANM and CRA.

		IMP3 expression, n		
			
Tissue	Cases, n	−	+/++	P-value
ANM	186	164	22	<0.001
CRA	186	43	143	

IMP3, insulin-like growth factor-II mRNA-binding protein 3; ANM, adjacent normal mucosa; CRA, colorectal adenocarcinoma; −, lack of IMP3 staining; +/++, weak/strong IMP3 staining.

**Table II:** Correlation between IMP3 protein and clinicopathological factors in CRA.

		IMP3 expression, n			
				
Parameters	Cases, n	−	+/++	χ^2^	P-value
Age (years)				1.073	0.302
>59	82	16	66		
≤59	104	27	77		
Gender				0.034	0.854
Male	119	27	92		
Female	67	16	51		
Histological grade				3.176	0.076
Well-differentiated	99	28	71		
Moderately/poorly-differentiated	87	15	72		
Tumor size (cm)				1.494	0.223
≤5	115	30	85		
>5	71	13	58		
Lymphoid metastasis				9.430	0.002
Present	75	26	49		
Absent	111	17	94		
Clinical stage				10.713	0.001
I–II	65	24	41		
III–IV	121	19	102		
CEA				1.473	0.226
Normal	76	21	55		
Increased	110	22	88		

IMP3, insulin-like growth factor-II mRNA-binding protein 3; CRA, colorectal adenocarcinoma; CEA, carcinoembryonic antigen level; −, no IMP3 staining; +/++, weak/strong IMP3 staining.

**Table III:** Correlation between IMP3 protein and Ki-67 in CRA.

	IMP3, n				
				
Immunostaining	−	+/++	n	r	P-value
Ki-67					
−	26	58	84		
+	17	85	102	0.169	0.021

IMP3, insulin-like growth factor-II mRNA-binding protein 3; CRA, colorectal adenocarcinoma; IMP3 −, lack of IMP3 staining; IMP3 +/++, weak/strong IMP3 staining; Ki 67 −, none or ≤5% postive cells; Ki 67 +, >5% positive cells.
